# Cytokines IL-1β and IL-10 are required for Müller glia proliferation following light damage in the adult zebrafish retina

**DOI:** 10.3389/fcell.2024.1406330

**Published:** 2024-06-13

**Authors:** Celine Lu, David R. Hyde

**Affiliations:** Department of Biological Sciences, Center for Stem Cells and Regenerative Medicine, and Center for Zebrafish Research, Galvin Life Sciences Building, University of Notre Dame, Notre Dame, IN, United States

**Keywords:** zebrafish, retina, regeneration, Müller glia, microglia, cytokines, IL-1β, IL-10

## Abstract

Zebrafish possess the ability to regenerate dying neurons in response to retinal injury, with both Müller glia and microglia playing integral roles in this response. Resident Müller glia respond to damage by reprogramming and undergoing an asymmetric cell division to generate a neuronal progenitor cell, which continues to proliferate and differentiate into the lost neurons. In contrast, microglia become reactive, phagocytose dying cells, and release inflammatory signals into the surrounding tissue following damage. In recent years, there has been increased attention on elucidating the role that microglia play in regulating retinal regeneration. Here we demonstrate that inflammatory cytokines are differentially expressed during retinal regeneration, with the expression of a subset of pro-inflammatory cytokine genes upregulated shortly after light damage and the expression of a different subset of cytokine genes subsequently increasing. We demonstrate that both cytokine IL-1β and IL-10 are essential for Müller glia proliferation in the light-damaged retina. While IL-1β is sufficient to induce Müller glia proliferation in an undamaged retina, expression of IL-10 in undamaged retinas only induces Müller glia to express gliotic markers. Together, these findings demonstrate the essential role of inflammatory cytokines IL-1β and IL-10 on Müller glia proliferation following light damage in adult zebrafish.

## 1 Introduction

Zebrafish are a widely recognized model organism in the vertebrate vision research field, with a highly conserved retinal structure and common cell types shared between zebrafish and mammalian retinas (Goldsmith and Harris, 2003; Gestri et al., 2012; Malicki et al., 2016; Angueyra and Kindt, 2018). Zebrafish are also a valuable model for understanding human ocular diseases (Phillips and Westerfield, 2014; Richardson et al., 2017; Patton and Tobin, 2019; Rosa et al., 2023). Unlike mammals, however, zebrafish have a remarkable capability to regenerate retinal neurons following retinal damage. Acute retinal neuronal loss induces resident Müller glia to reprogram and undergo an asymmetric cell division to produce a neuronal progenitor cell (NPC; Bernardos et al., 2007; Gorsuch and Hyde, 2014; Lahne et al., 2020; Powell et al., 2016). The NPCs continue to proliferate and migrate to the proper retinal layer, where they differentiate into the missing retinal neurons (Gemberling et al., 2013; Lenkowski and Raymond, 2014; Lahne et al., 2021). This mechanism enables the zebrafish retina to not only regenerate dying retinal neurons, but ultimately restore lost visual responses (Sherpa et al., 2008; 2014). A large number of studies have identified genes that are expressed within the Müller glia that are required for Müller glia reprogramming and reentry into the cell cycle (Hoang et al., 2020; Lahne et al., 2020). However, much less is known about extrinsic signals that regulate Müller glia-dependent neuronal regeneration in the adult retina, Recently, there has been increasing interest in the role of microglia in regulating neuronal regeneration following retinal damage in zebrafish (White et al., 2017; Mitchell et al., 2019; Van Dyck et al., 2021; Iribarne and Hyde, 2022). Microglia are the resident immune cells of the nervous system and play a vital role during neurogenesis, synaptic maintenance, and surveillance for abnormalities (Paolicelli et al., 2011; Parkhurst et al., 2013; Gogoleva et al., 2019; Casali and Reed-Geaghan, 2021; Wang and Li, 2021). Under pathological insults, microglia become activated and phagocytose dying cells and release inflammatory signals within the surrounding tissue (Fricker et al., 2012; Tanaka et al., 2013; Fu et al., 2014; Wolf et al., 2017; Muzio et al., 2021). In adult zebrafish, retinal damage induces resident microglia and infiltrating macrophages to proliferate and migrate to the site of damage, where they actively phagocytose cellular debris (Mitchell et al., 2018). In addition, microglia are essential to properly regulate the Müller glia-dependent neuronal regeneration response. Depleting microglia, either by pharmacological treatment or ablation prior to neuronal damage, inhibits Müller glia reprogramming and proliferation following retinal damage (White et al., 2017; Conedera et al., 2019; Zhang et al., 2020; Van Dyck et al., 2021; Iribarne and Hyde, 2022). This reduced regenerative response by the Müller glia is likely due to the loss of secreted signals from the depleted microglia to the Müller glia.

Activated microglia can release both pro- and anti-inflammatory cytokines in response to neuronal damage (Frank et al., 2007; Rodríguez-Gómez et al., 2020; Jesudasan et al., 2021). Cytokines that commonly exert a pro-inflammatory effect such as IL-1β, IL-6, and Ifng1 induce inflammation, which is intended to prevent further neuronal damage (Frank et al., 2007; Smith et al., 2012; Rodríguez-Gómez et al., 2020; Jesudasan et al., 2021). However, prolonged and excessive exposure to these cytokines can be detrimental due to their inherent neurotoxicity. In the adult zebrafish retina, one pro-inflammatory cytokine, TNFα, was shown to be necessary and sufficient to induce the Müller glia-dependent neuronal regeneration response (Nelson et al., 2013; Conner et al., 2014). However, TNFα is not initially produced by the microglia, rather it is released from the dying retinal neurons (Nelson et al., 2013). In contrast, cytokines that are often associated with an anti-inflammatory effect, such as IL-4, IL-10, and IL-13, attenuate the inflammatory response over time to focus on tissue repair and homeostasis (Lively et al., 2018; Sun et al., 2019; Bottiglione et al., 2020; Miao et al., 2020; Shemer et al., 2020; Chen et al., 2022). A successful tissue recovery is achieved only when the appropriate balance of inflammatory cytokines is expressed (Cicchese et al., 2018; Soliman and Barreda, 2023).

In this manuscript, we explored the expression and role of the inflammatory response following light damage of the zebrafish retina. We confirmed that a subset of inflammatory cytokines increased shortly after retinal damage, followed by increased expression of three cytokines that possess anti-inflammatory effects. We then demonstrated that the pro-inflammatory cytokine IL-1β is both necessary and sufficient for Müller glia proliferation. Additionally, IL-1β expression induced the expression of cytokine IL-10. We then demonstrated that IL-10 was also required for Müller glia proliferation in the light-damaged retina but was not sufficient to induce Müller glia proliferation in undamaged retinas. However, in the absence of retinal damage, IL-10 induced Müller glia to exhibit a hypertrophic morphology and express several gliotic genes, which are essential for Müller glia reprogramming (Thomas et al., 2016; Hoang et al., 2020). This work demonstrates that the dynamic expression of at least two inflammatory cytokines is essential to induce Müller glia proliferation at the outset of retinal regeneration in zebrafish.

## 2 Materials and methods

### 2.1 Fish maintenance

Female and male zebrafish (*Danio rerio*) were used in this study and maintained at 28°C under normal light conditions (14 h light:10 h dark) in the Freimann Life Science Center at the University of Notre Dame as described previously (Vihtelic and Hyde, 2000). Fish were 6–12 months old and 4–5 cm in length. The fish lines utilized in this study include *albino,* and *albino;Tg*(*gfap:EGFP*)^*nt11*^. All animal care protocols were approved by the University of Notre Dame Animal Care and Use Committee and in compliance with the Association of Research in Vision and Ophthalmology for the use of animal in vision research.

### 2.2 Light damage paradigm

Adult *albino* and *albino;Tg*(*gfap:EGFP*)^*nt11*^ zebrafish were dark-adapted for 2 weeks and exposed to constant intense light for up to 96 h as previously described (Vihtelic and Hyde, 2000; Kassen et al., 2006; Lahne et al., 2021). The temperature of the tanks was maintained at 32°C, which is optimal to induce maximal photoreceptor cell death. Fish were euthanized in a 1:500 dilution of 2-phenoxyethanol (2-PE; 77699; Sigma-Aldrich, St. Louis, MO) in system water after each collection timepoint.

### 2.3 Protein and drug injections

#### 2.3.1 Injection of microglia drugs pexidartinib and dexamethasone

To inhibit microglia during light treatment, a combined treatment of Pexidartinib, also known as PLX3397, (PLX; S78181; Selleck Chemicals, Houston, TX) and Dexamethasone (Dex; D1756; Sigma-Aldrich) were used. PLX3397 reduces the number of microglia and Dexamethasone will reduce the inflammatory response of any remaining microglia. Dark-adapted *albino:Tg*(*gfap:EGFP*)^*nt11*^ fish were anesthetized in 1:1000 2-PE and a small incision was made in the cornea with a double-edged sapphire blade (504077; World Precision Instruments, Sarasota, FL). Zebrafish were then intravitreally injected with either 0.5 μL of DMSO (vehicle control) or a combination of 250 µM of Pexidartinib and 250 µM of Dexamethasone using a 2.5 μL Hamilton syringe with 33-gauge rounded needle (7762-06; Hamilton, Reno, NV) every 24 h from the start of light treatment to 72 h of light treatment (LT). Fish were removed from either the constant light treatment room or the standard light treatment room for approximately 15 min to perform intravitreal injections and then returned to the corresponding light conditions.

#### 2.3.2 Intravitreal injection of caspase-1 inhibitor, Ac-YVAD-cmk

To investigate the role of inflammatory cytokine IL-1β, dark-adapted *albino;Tg*(*gfap:EGFP*)^*nt11*^ fish were anesthetized in 1:000 2-PE and intravitreally injected with 0.5 μL of either DMSO (vehicle control) or 1 mM of Ac-YVAD-cmk (YVAD; SML0429; Sigma-Aldrich). Fish were injected 24 h prior to the start of light treatment and subsequently every 24 h until 36 h LT. Injections were performed as described in Section 2.3.1.

#### 2.3.3 Injection of recombinant proteins IL-1β and IL-10

Adult *albino* and *albino;* Tg (*gfap:*EGFP)^nt11^ were used for undamaged retinal injections. Fish were anesthetized and intravitreally injected with 0.5 μL of either PBS (vehicle control), recombinant zebrafish IL-1β protein (AB236204; Abcam, Cambridge, United Kingdom) or IL-10 protein (RP1023Z; Kingfisher Biotech, St. Paul, MN) every 24 h. After each injection, fish were placed in a dark incubator at 32°C, which corresponds to the tank temperature that the light-treated fish are kept at and enables the initiation of maximal Müller glia and NPC proliferation in a short time frame (Conner et al., 2014). Fish were euthanized and collected at 2- and 3-days post injections (dpi). Injections were performed as described in Section 2.3.1.

### 2.4 Morpholino-mediated knockdown and electroporation

Morpholino oligos were synthesized by GeneTools LLC (Philomath, OR) and contained a positively charged lissamine tag. Dark-adapted *albino; Tg*(*gfap:EGFP*)^*nt11*^ zebrafish were intravitreally injected with 0.5 μL of either 1 mM Standard Control*, il-1β* or *il-10* translation-blocking morpholinos. Electroporation were then performed as previously described (Thummel et al., 2011; Campbell et al., 2021) and immediately placed in constant light treatment following electroporation. The following morpholinos were used: Standard Control: 5′-CCT​CTT​ACC​TCA​GTT​ACA​ATT​TAT​A-3’ (Nasevicius and Ekker, 2000), *il-1β*: 5′- CCC​ACA​AAC​TGC​AAA​ATA​TCA​GCT​T -3' (López-Muñoz et al., 2011; Frame et al., 2020; Wattrus et al., 2022), *il-10*: 5′- AAT​CAG​TGG​CAC​TTA​CGT​TTA​TGT​T- 3′, *tnfa*: 5′- AGC​TTC​ATA​ATT​GCT​GTA​TGT​CTT​A- 3’ (Nelson et al., 2013).

### 2.5 Immunohistochemistry

Immunohistochemistry was performed as previously described (Lahne et al., 2021; Boyd et al., 2023). Eyes were collected and fixed in a 9:1 ethanolic formaldehyde solution overnight at 4°C. Eyes were rehydrated in a series of ethanol washes and incubated in 30% sucrose overnight at 4°C. Eyes were then cryoprotected in a mixture of 2:1 Tissue Freezing Media:30% sucrose overnight at 4°C. Retinas were then frozen in Tissue Freezing Media, sectioned at 14 μm thickness, and stored at −80°C until immunostaining.

Slides were rehydrated in PBS for 30 min at room temperature and blocked in 2% DMSO, 2% normal goat serum, 1% Tween-20, and 0.4% Triton X in PBS for 1 h at room temperature. Primary antibodies diluted in blocking solution were applied on the slide and incubated overnight at room temperature. Primary antibodies used in this study included: chicken anti-GFP (1:1000; AB13970; Abcam), mouse anti-PCNA (1:1000; P8825; Sigma-Aldrich) and rabbit anti-Lcp1 (1:400; GTX134687; GeneTex, Irvine, CA). Slides were washed with PBS/0.05% Tween-20 (PBS-T) and incubated in secondary antibodies diluted in blocking solution for 1 h at room temperature. Fluorescent-tagged secondary antibodies (Life Technologies, Carlsbad, CA) used in this study included: Alexa Fluor goat anti-chicken 488 (1:1000; A11039), Alexa Fluor goat anti-mouse 594 (1:1000; A11032), and Alexa Fluor goat anti-rabbit 647 (1:1000; A21245). 4,6-diamidino-2phenylindol (DAPI; 1:1000; D1306, Thermo Fisher Scientific; Waltham, MA) was also applied for nuclear localization. Slides were washed in PBS-T and mounted in Prolong Gold Antifade Reagent (P36930; Life Technologies).

### 2.6 RNA isolation and quantitative real-time polymerase chain reaction

Dorsal retinas from adult *albino* zebrafish were isolated at specific time points of constant light and RNA isolation was performed with Trizol (15596-026; Thermo Fisher) as previously described (Campbell et al., 2021; Lahne et al., 2021). For each time point, 6-7 dorsal retinas were collected and RNA concentrations were measured. The cDNA samples were synthesized using LunaScript RT SuperMix kit (E3101; New England Biolabs, Ipswich, MA) and stored at −80°C. Sample reactions were assembled using Luna universal qPCR Master Mix (M3003, New England Biolabs) following the manufacturer’s instructions with TaqMan primers listed in Table 1 (Applied Biosystem). StepOnePlus Real-Time PCR system (4276600; Thermo Fisher) was used to perform qRT-PCR reactions with the following conditions: 1 min at 95°C, followed by 40 cycles of 15 s at 95°C and 30 s at 60°C. qRT-PCR reactions were analyzed using the comparative ΔΔCT method as previously described (Gorsuch et al., 2017; Lahne et al., 2021) using 18 s rRNA as the reference gene. The CT values for 18 s rRNA are consistent across all rounds of qRT-PCR experiments. Light-treated retinas used 0 h LT to generate a log_2_-fold change in gene expression levels (Kassen et al., 2006) and undamaged retinas used PBS (vehicle) control to generate a log_2_-fold change in gene expression levels. Three biological replicates were conducted with each sample.

**TABLE 1:** Primer information.

Gene	Primer sequence
*il-1β-*F	5′- GCT​CAT​GGC​GAA​CGT​CAT​CC-3′
*il-1β-*R	5′- CGC​ACT​TTC​AAG​TCG​CTG​CT-3′
*il-6-*F	5′-ACA​CTC​AGA​GAC​GAG​CAG​TTT​G-3′
*il-6*-R	5′-ACC​ACG​TCA​GGA​CGC​TGT​AG-3′
*ifng1*-F	5′-CGC​ATG​CAG​AAT​GAC​AGC​GT-3′
*ifng1-*R	5′- ACA​AAG​CCT​TTC​GCT​GGA​CG-3′
*il-10*-F	5′-GCA​CTC​CAC​AAC​CCC​AAT​CG-3′
*il-10-*R	5′-TGG​CAA​GAA​AAG​TAC​CTC​TTG​CAT-3′
*il-4-*F	5′-GCA​GCA​TAT​ACC​GGG​ACT​GG-3′
*il-4-*R	5′-TGG​CAG​CAT​GCT​TTG​GTT​TTT-3′
*il-13-*F	5′-AAG​GAA​GTG​GCC​TGA​AGT​GTG​A-3′
*il-13-*R	5′-TTC​TTG​TCG​GTA​CGG​AAA​GGG​T-3′
*mpeg-F*	5′-CAC​AGA​AAA​CCA​GCG​CAT​GAA-3′
*mpeg-R*	5′-CAG​ATG​GTT​ACG​GAC​TTG​AAC​CC-3′
*gfap-*F	5′- ACT​CAA​TGC​TGG​CAA​AGC​CC-3′
*gfap*-R	5′- CCG​CTT​CAT​CCA​CAT​CTT​GTC​TG-3′
*yap*-F	5′- TGA​GAT​GGA​GAC​AGG​TGA​C-3′
*yap-*R	5′- ATG​GCG​TCT​AGG​TAA​TCG​G-3′
*glula-F*	5′- GGC​AAC​TGG​AAT​GGT​GCT​GG-3′
*glula*-R	5′- AGC​ATT​GTC​CAG​GCC​TCC​TT-3′
*glulb-*F	5′- GCC​TGT​CTG​TAT​GCT​GGG​GT-3′
*glulb*-R	5′- CCT​GTG​TAG​GAG​GAA​GCG​GG-3′
*manf*-F	5′- TGG​AGA​GTG​TGA​AGT​CTG​TGT​G-3′
*manf-*R	5′- GCT​GCA​TCA​CTC​GTT​GCA​C-3′
*vim-*F	5′- TGA​GAT​CGC​CAC​CTA​CAG​GA-3′
*vim*-R	5′- CCT​TCA​TGG​ACT​CTC​GCA​GG-3′
*nes-*F	5′- GCT​TCA​ACA​TCT​TCA​GGC​CC-3′
*nes-*R	5′- CTG​TCG​ATT​CTC​AGG​CCC​TC-3′
*aqp4*-F	5′- CCA​TCT​CTT​TGC​GAT​CCC​GT-3′
*aqp4-*R	5′- TTC​AGG​TCA​GGG​TCA​GGA​CA-3′
*rhod*-F	5′- GCT​GAG​CGC​CAC​ATC​CA-3′
*rhod*-R	5′- AGG​CAC​GTA​GAA​TGC​CGG-3′
*pcna*-F	5′-TAC​TCA​GTG​TCT​GCT​GTG​GTT​TCC-3′
*pcna*-R	5′-CAT​TTA​ATA​AGT​GCG​CCC​GC-3′
*18s*-F	5′-TCG​GCT​ACC​ACA​TCC​AAG​GAA​GGC​AGC-3′
*18s*-R	5′-TTG​CTG​GAA​TTA​CCG​CGG​CTG​CTG​G CA-3′

### 2.7 RNAscope *in situ* hybridization

RNAscope Multiplex Fluorescent v2 Assay (Advance Cell Diagnostics; Newark, CA) was utilized for RNA *in situ* hybridization. Sample fixation and immunohistochemistry with *in situ* hybridization were performed according to a protocol described previously (Campbell et al., 2021; Boyd et al., 2023). Briefly, frozen tissue sections were washed in PBS for 5 min before baking at 60°C for 1 h. Sections were then post-fixed in 4% paraformaldehyde at room temperature for 1 h. Sections were dehydrated in sequential 50%, 70%, 100% ethanol washes for 5 min each and twice in 100% before baking at 60°C for 1 h. Slides were given a hydrogen peroxide treatment provided by the manufacturer (Advance Cell Diagnostic) at room temperature for 1 h. The slides were washed in distilled water and immediately submerged into a mildly boiling Target Retrieval Reagent solution (Advance Cell Diagnostic) for 15 min. Sections were placed in distilled water at room temperature and dehydrated in 100% ethanol. A hydrophobic barrier (ImmEdge Hydrophobic pen; H-4000; Vector Laboratories; Burlingame, CA) was applied on the slides and baked at 60°C for 1 h before drying overnight at room temperature.

Sections were given a Protease III treatment at 40°C for 30 min in the HybEZ™ Oven (Advance Cell Diagnostic). Slides were washed with distilled water followed by probe hybridization at 40°C for 2 h. The probe used in this study was Dr-*il1b*-C1 (432971; Advance Cell Diagnostic), which was taken from NCBI accession of *D. rerio il-1β*: NM_212,844.2. A 3-plex negative control probe, provided by the manufacturer, was applied simultaneously with experiment slides. Following probe hybridization, sections were incubated with a probe amplification AMP1 solution at 40°C for 30 min followed by a buffer wash. Signal development with HRP was based on the manufacturer’s protocol with Opal 570 (1:1000; FP1488001KT; Akoya Biosciences; Menlo Park, CA). Once signal development was complete, slides were washed in PBS-T for 5 min at room temperature before proceeding with immunohistochemistry. Primary antibodies used were chicken anti-GFP (1:1000; AB13970; Abcam) and rabbit anti-Lcp1 (1:400, GTX134697; Genetex, Irvine, CA). Fluorescent-tagged secondary antibodies used were Alexa Fluor goat anti-chicken 488 (1:1000; A11039; Thermo Fisher Scientific) and Alexa Fluor goat anti-rabbit 647 (1:1000; A21245; Thermo Fisher Scientific) and DAPI (1:1000; D1306; Thermo Fisher Scientific) was applied to stain nuclei.

### 2.8 Image acquisition and data analysis

A Nikon A1 confocal microscope and Leica Stellaris eight confocal microscope with a 40x oil-immersion objective were used to acquire approximately 10 µm z-stack with 1.0 µm step size images of the central dorsal retina. Cell counts were manually performed using FIJI/ImageJ software and normalized to a 300 µm length of the retina. Scale bars for images are either 20, 10, or 5 μm and are indicated in the figures and figure legends.

### 2.9 Single-cell RNA-Seq analysis

We used single-cell RNA-Seq (scRNA-Seq) data from whole-light-damaged retinas that was previously published (Hoang et al., 2020). scRNA-seq analysis was performed using Seurat (Hao et al., 2021). Violin plots were generated using cell clusters identified and maintained from Hoang et al. (2020). Differential expression analyses were performed with microglia and Müller glia.

### 2.10 Statistical analyses

The data in this study were analyzed with at least three independent trials. The sample size and the mean ± SEM are stated in the text and each figure legend. Statistical analysis was performed using GraphPad Prism 10 (San Diego, CA). Statistical significance was determined using either a Student’s t-test, a one-way ANOVA followed by Bonferroni’s *pos-hoc* test, or a two-way ANOVA followed by Bonferroni’s *post hoc* test. A *p*-value less than 0.05 indicated statistical significance, with graphs displaying * for *p* ≤ 0.05, ** for *p* ≤ 0.01, *** for *p* ≤ 0.001, **** for *p* ≤ 0.0001, and n. s. for no significance.

## 3 Results

### 3.1 Cotreatment with pexidartinib and dexamethasone reduces the number of microglia and proliferating Müller glia in light-damaged retinas

**FIGURE 1:**
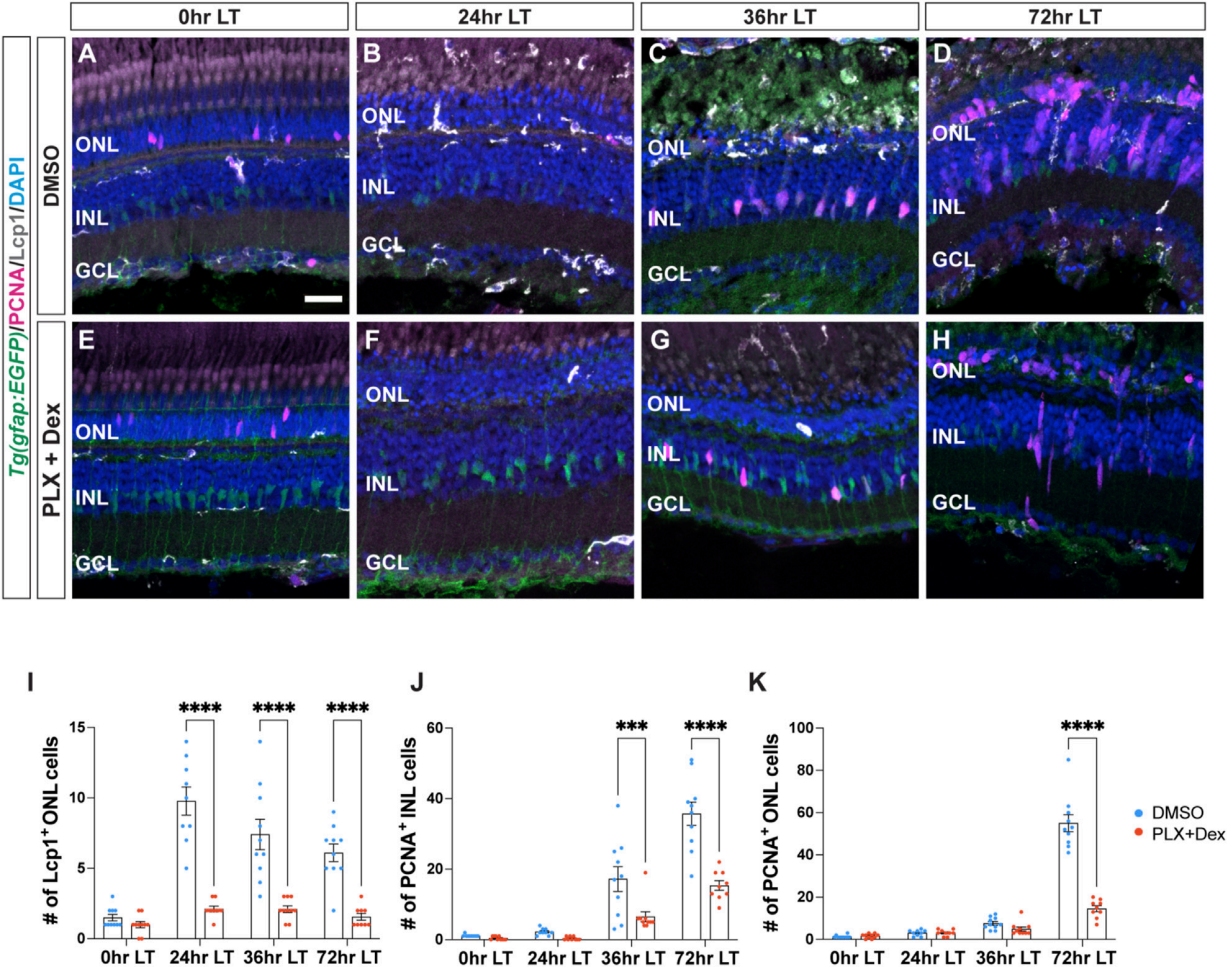
Treatment with PLX3397 and Dexamethasone reduces the number of microglia and proliferating Müller glia in light-damaged retinas. **(A–H)** Confocal images of *albino;Tg*(*gfap:EGFP*)^*nt11*^ retinas that were light-damaged and collected at 0, 24, 36, and 72 h of light treatment (LT). Eyes were intravitreally injected with either DMSO **(A–D)** or PLX3397 and Dexamethasone (PLX + Dex, E-H) at 0 h LT. Sections were immunostained to detect GFP (Müller glia, green), PCNA (proliferating cells, magenta), and Lcp1 (microglia, white), with DAPI counterstain (nuclei, blue). **(I)** Quantifications of the numbers of Lcp1^+^ ONL cells under the different conditions described above. **(J,K)** The numbers of PCNA^+^ cells were quantified in the INL **(J)** and ONL **(K)**. Quantifications were normalized to 300 μm along the length of the central-dorsal retina. Statistical analyses were performed using two-way ANOVA with Bonferroni’s *post hoc* test. Graphs represent the Mean ± SEM and n ≥ 9. ***, *p* < 0.001, ****, *p* < 0.0001. ONL, outer nuclear layer; INL, inner nuclear layer; GCL, ganglion cell layer. Scale bar in A is 20 µm and is the same for **(B–H)**.

It was previously demonstrated that microglia are required for Müller glia proliferation following retinal damage (White et al., 2017; Conedera et al., 2019; Silva et al., 2020; Iribarne and Hyde, 2022). To confirm that microglia are required for retinal regeneration in light-damaged adult zebrafish retinas, we utilized two pharmacological treatments to reduce the number of activated microglia. Pexidartinib, or PLX3397 (PLX), is a small molecule that targets the colony-stimulating factor 1 receptor (CSF1R), which blocks CSF1 binding, a regulator of microglia and macrophage survival, production, and differentiation (Elmore et al., 2014; Conedera et al., 2019; Han et al., 2022). Dexamethasone (Dex) is an anti-inflammatory glucocorticoid that has immunosuppressive effects on microglia (Park et al., 2019; Hui et al., 2020; Iribarne and Hyde, 2022). Together, these drugs should reduce the number of microglia and the inflammatory response of any remaining microglia. Dark-adapted *albino;Tg*(*gfap:EGFP*)^*nt11*^ zebrafish were intravitreally injected with a combination of PLX and Dex every 24 h until 72 h of constant light treatment (LT). Retinas were collected at 0, 24, 36, and 72 h LT and immunolabeled for GFP and PCNA to label proliferating Müller glia and Lcp1, a marker commonly used for microglia, macrophages, and other leukocyte cells (Figures 1A–H). Because macrophages and other leukocyte cells are typically uncommon in the retina, unless the blood-brain barrier is compromised, Lcp1^+^ retinal cells are predominantly microglia.

The PLX + Dex coinjected retinas possessed significantly fewer Lcp1^+^ cells compared to the DMSO (vehicle) control group at all time points of constant light (Figure 1I; DMSO: 24 h LT: 9.78 ± 0.99, 36 h LT: 7.40 ± 1.08, 72 h LT: 6.10 ± 0.62; PLX + Dex: 24 h LT: 2.11 ± 0.20, *p* < 0.0001, 36 h LT: 2.10 ± 0.23, *p* < 0.0001, 72 h LT: 1.56 ± 0.24, *p* < 0.0001). The reduced number of microglia during LT also correlated with significantly fewer PCNA^+^ Müller glia in the INL at 36 h LT (Figure 1J; DMSO: 17.6 ± 3.50; PLX + Dex: 6.50 ± 1.41, *p* = 0.0005). At 72 h LT, when Müller glia-derived neuronal progenitor cells (NPCs) are present in both the INL and ONL, there were significantly fewer proliferating NPCs in the drug-treated retinas relative to the controls (Figure 1K; DMSO INL: 35.70 ± 3.29, ONL: 55.00 ± 3.98; PLX + Dex INL: 15.33 ± 1.36, *p* < 0.0001, ONL: 14.56 ± 1.42, *p* < 0.0001). These findings suggest that the combined treatment of PLX and Dex effectively reduced the number of microglia and proliferating Müller glia in light-damaged retinas.

### 3.2 Cytokines are differentially expressed following light damage

**FIGURE 2:**
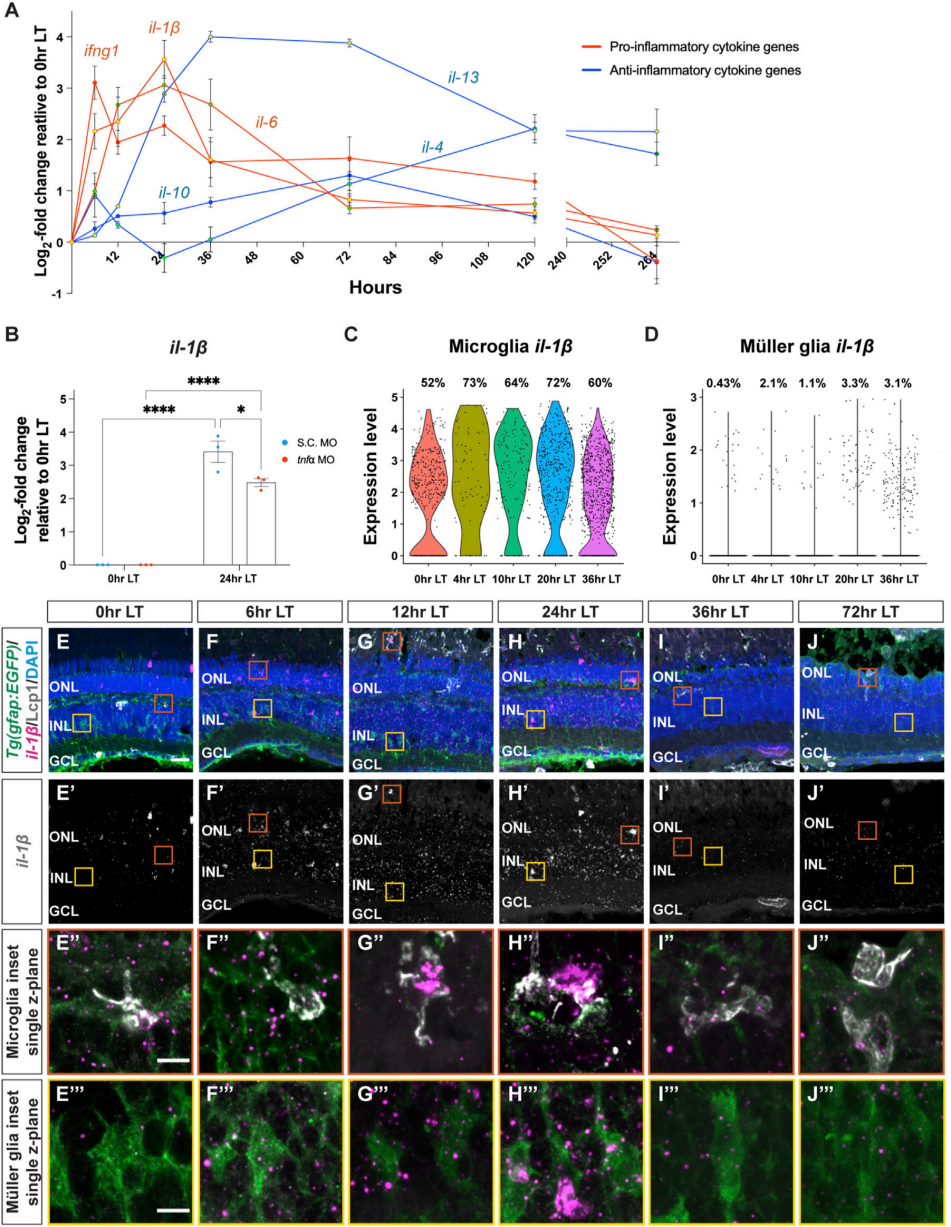
Cytokine genes are dynamically expressed following light damage. **(A)** qRT-PCR analysis of three cytokine genes, which are commonly associated with inflammatory effects: *il-1β* (red line, yellow dot)*, il-6* (red line, green dot)*,* and *ifng1* (red line, red dot) and three cytokine genes, which are usually associated with inhibiting inflammation: *il-10* (blue line, blue dot), *il-13* (blue line, yellow dot)*, il-4* (blue line, green dot)*,* across light treatment (LT) time course (0, 6, 12, 24, 36, and 72 h LT) and 1-day (120 h) and 7-days (264 h) recovery (after completing 96 h constant LT). All values are normalized to 18 s rRNA reference gene. The mRNA expression levels are displayed as log_2_-fold change relative to 0 h LT from three independent replicates with a pool of 6-7 dorsal retinas for each replicate. **(B)** qRT-PCR analysis of *il-1β* expression at 0 h and 24 h LT in Standard Control morphants (S.C. MO, blue circles) and *tnfa* morphants (red circles). Data was normalized to 18 s rRNA reference gene and displayed as log_2-_fold change relative to the S.C. MO control group. For the qRT-PCR, three independent replicates were performed with a pool of 6-7 dorsal retinas for each replicate. **(C,D)** Previously published single-cell RNA-Seq data (Hoang et al., 2020) were analyzed by violin plots for *il-1β* expression in microglia **(C)** and Müller glia **(D)**. The time points included in the snRNA-Seq dataset correspond to 0, 4, 10, 20, and 36 h LT. (E–J‴) Confocal images of dark-adapted *albino;Tg*(*gfap:EGFP*)^*nt11*^ zebrafish retinas that were isolated at 0 **(E–E’’’)**, 6 **(F–F’’’)**, 12 **(G–G’’’)**, 24 **(H–H’’’)**, 36 **(I–I’’’)**, and 72 h LT **(J–J’’’)**. Retinal sections were labeled by *in situ* hybridization with a probe for *il-1β* magenta: **(E–J,E’’–J’’’)**, grayscale: **(E’–J’)**. Sections were also immunostained to detect GFP Müller glia, green: **(E–J,E’’-J’’’)** and Lcp1 microglia, grayscale: **(E–J, E’’–J’’’)**, with DAPI counterstain nuclei, blue: **(E–J, E′′–J‴)**. Orange boxes in **(E–J)** were magnified to better portray *il-1β* signal in microglia **(E’’–J’’)** and yellow boxes in E-J were magnified to better portray *il-1β* signal in Müller glia **(E’’’–J’’’)**. The magnified images in **(E’’–J’’’)** represent single z-plane images. ONL, outer nuclear layer; INL, inner nuclear layer; GCL, ganglion cell layer. Scale bar in E is 20 µm and is the same for F-J’ and scale bars in E’’ and E’’’ are 5 µm and are the same for **(F’’–J’’)** and **(F’’’–J’’’)**, respectively. Graphs represent the Mean ± SEM and *n* = 3. *, *p* < 0.05, ****, *p* < 0.0001.

In pathological conditions, activated microglia play a critical role in regulating inflammatory signaling, which attracts other cells to the site of injury and modulates the surrounding microenvironment (Streit et al., 2004; Bosak et al., 2018; Mitchell et al., 2018; Iribarne and Hyde, 2022). To examine the expression of a subset of inflammatory cytokines during LT, we used quantitative real-time PCR (qRT-PCR). Dark-adapted *albino* zebrafish were light-damaged, and retinas were collected and RNA isolated at 0, 6, 12, 24, 36, and 72 h LT, as well as 1-day (120 h) and 7-days (264 h) after terminating the constant light treatment at 96 h LT. These time points were selected based on established events observed during light-induced damage and regeneration of the adult zebrafish retina, including: 1) photoreceptor cell death between 12 and 24 h, 2) Müller glia proliferation 31–36 h, and 3) amplification and migration of NPCs to the ONL between 68 and 96 h (Vihtelic and Hyde, 2000; Gorsuch and Hyde, 2014). Two additional time points were included: 1-day (120 h) and 7-days (264 h) after terminating the constant light treatment at 96 h LT, which represent time points that correspond to the regeneration of retinal neurons (Lyu et al., 2023). We investigated the temporal expression of three cytokine genes commonly associated with pro-inflammatory effects: *interleukin-1β* (*il-1β*), *interleukin-6* (*il-6*), and *interferon gamma one* (*ifng1*) and three cytokine genes usually associated with anti-inflammatory effects: *interleukin-4* (*il-4*), *interleukin-10* (*il-10*), and *interleukin-13* (*il-13*). The expression of all three cytokine genes in the first group rapidly increased during the first 24 h after initiating the LT and then decreased over the next several days (Figure 2A). In contrast, the three cytokine genes in the latter group peaked in their expressions between 72 and 120 h after the start of constant light. (Figure 2A). Thus, these six cytokines exhibit temporally distinct expression profiles following light damage.

We previously demonstrated that *tumor necrosis factor-alpha* (*tnfa*) expression increases in damaged/dying retinal neurons and is required and sufficient to induce Müller glia proliferation (Nelson et al., 2013). We hypothesized that TNFα expression from the dying photoreceptors might induce *il-1β* in the microglia. To test this, we intravitreally injected and electroporated either the Standard Control morpholino (S.C. MO), which is not complementary to any known sequence in the zebrafish genome, or the *tnfa* MO (Nelson et al., 2013) into dark-adapted *albino;Tg*(*gfap:EGFP*)^*nt11*^ zebrafish. Fish were then placed in constant bright light for either 0 h or 24 h and then retinas were collected, mRNA was purified from dorsal retinas, and subjected to qRT-PCR using primers for the *il-1β* mRNA. As expected, there was a significant increase in *il-1β* expression at 24 h LT relative to 0 h LT (Figure 2B). In addition, the *tnfa* morphant expressed significantly lower amounts of *il-1β* relative to the S.C. morphant (Figure 2B). This is consistent with TNFα being required to induce the increased expression of *il-1β* in the light-damaged retina.

We analyzed *il-1β* further because it had the highest level of expression of the three early onset expressing cytokines that we tested. To identify what cells expressed *il-1β* during constant light treatment, we analyzed *il-1β* expression in our previously published scRNA-Seq dataset (Hoang et al., 2020). The time points that were analyzed in this dataset were 0, 4, 10, 20, and 36 h LT. Expression of *il-1β* increased rapidly, peaking at 20 h LT and gradually declining (Figure 2C). Within this dataset of microglia from 0 to 36 h LT, the average *il-1β* expression peaked at 4 h LT (2.20), as did the percentage of microglia expressing *il-1β* (Figure 2C, 73%)*.* We then examined if the Müller glia expressed *il-1β* and found that, while *il-1β* expression was significantly lower in the Müller glia relative to the microglia, the average *il-1β* expression peaked at 36 h LT (0.044) and the percentage Müller glia expressing *il-1β* peaked at 20 h LT (Figure 2D, 3.3%). Thus, *il-1β* expression is rapidly expressed in microglia after the start of constant light damage and then followed by expression in the Müller glia.

To confirm the spatial pattern of *il-1β* expression during LT, we utilized RNAscope *in situ* hybridization with immunohistochemistry (Figures 2E–J’’’). Low levels of *il-1β* probe signal were present at 0 h and increased through 24 h LT (Figures 2E’–H’), before decreasing in expression at 36 and 72 h LT (Figures 2I’, J’), consistent with the qRT-PCR results. Over the time course, the *il-1β* probe colocalized with both the Lcp1^+^ microglia (Figures 2E’’–J’’) and GFAP^+^ Müller glia cell populations (Figures 2E’’’–J’’’). Taken together, these data reveal that inflammatory cytokines are expressed in a dynamic temporal manner during LT, and the pro-inflammatory cytokine gene *il-1β* is expressed sequentially in both microglia and Müller glia.

### 3.3 Pro-inflammatory cytokine IL-1β is both necessary and sufficient for Müller glia proliferation in light-damaged retinas

**FIGURE 3:**
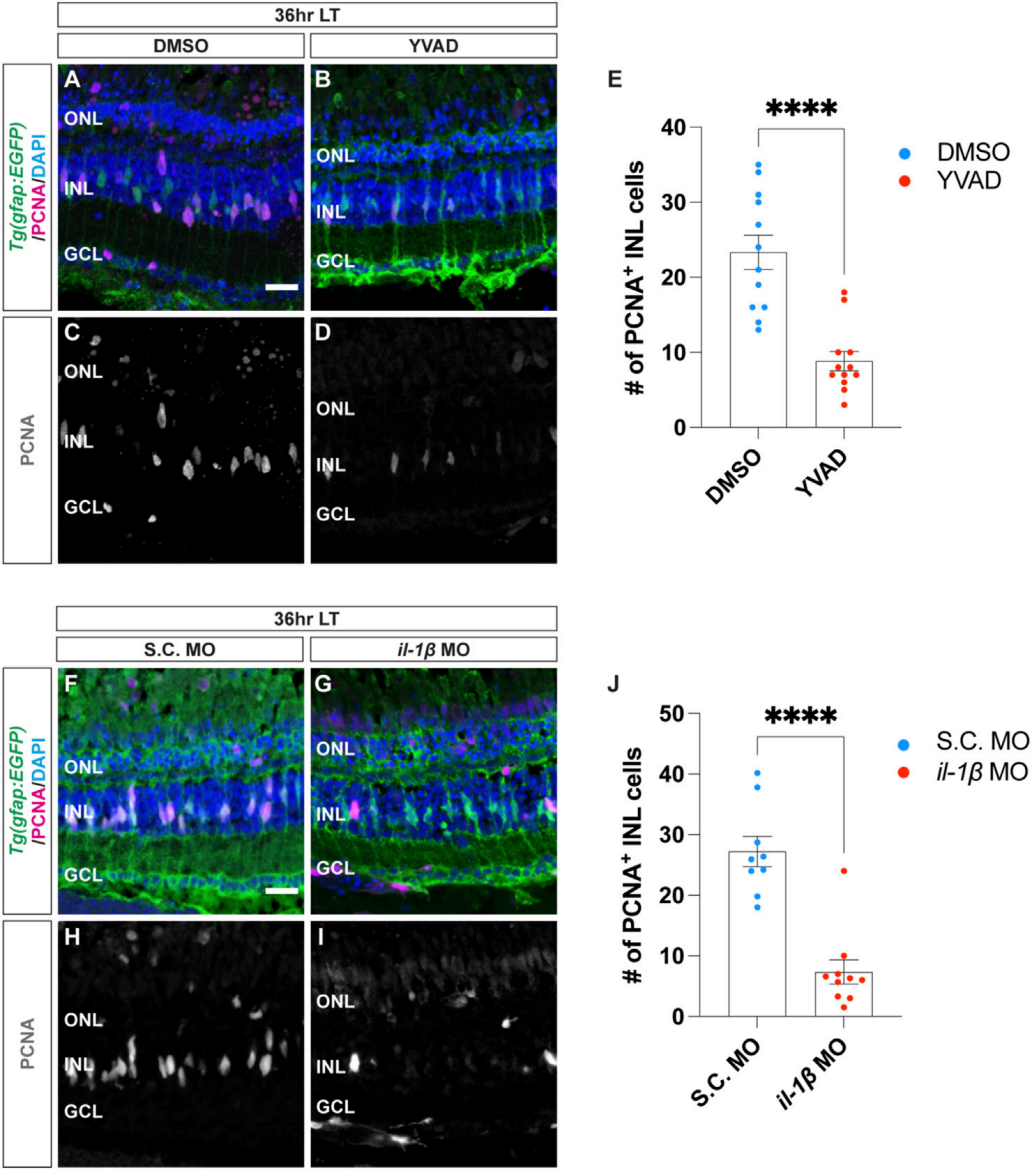
Cytokine IL-1β is necessary for Müller glia proliferation in light-damaged retinas. **(A–D)** Confocal images from *albino;Tg*(*gfap:EGFP*)^*nt11*^ retinas that were intravitreally injected with a caspase-1 inhibitor Ac-YVAD-cmk (YVAD) 24 h prior to the start of light treatment and continued to inject every 24 h until 36 h LT. At 0 h LT, retinas were intravitreally injected with either DMSO vehicle control: **(A,C)** or YVAD **(B,D)**. Retinal sections were immunostained to detect GFP Müller glia, green: **(A,B)** ande PCNA proliferating cells, magenta in **(A,B)**, grayscale in **(C,D)**, with DAPI counterstain (nuclei, blue: **(A,B)**. **(E)** The numbers of PCNA^+^ INL cells in the YVAD group (red circles) relative to the DMSO control group (blue circles). **(F–I)** Confocal images of retinas from *albino;Tg*(*gfap:EGFP*)^*nt11*^ zebrafish electroporated with either Standard Control (S.C.; **(F,H)** or *il-1β* morpholino (MO; **(G,I)** to knockdown IL-1β protein expression and immediately placed in constant light treatment. Retinas were collected at 36 h LT and immunostained to detect GFP Müller glia, green: **(F,G)** and PCNA proliferating cells, magenta in **(F,G)**, grayscale in **(H,I)**, with DAPI counterstain (nuclei, blue: **(F,G)**. **(J)** Quantification of the numbers of PNCA^+^ INL cells in the *il-1β* morphant (red circles) and the S.C. MO (blue circles). Quantifications were normalized to 300 μm along the length of the central-dorsal retina. Statistical analyses were performed using Student’s t-test. Graphs represent the Mean ± SEM and n ≥ 7. ****, *p* < 0.0001. ONL, outer nuclear layer; INL, inner nuclear layer; GCL, ganglion cell layer. Scale bars in **(A,F)** are 20 μm and are the same for **(B–D)** and **(G–I)**, respectively.

To investigate how pro-inflammatory cytokine IL-1β may affect Müller glia proliferation following light damage, we inhibited IL-1β secretion by using caspase-1 inhibitor, Ac-YVAD-cmk (YVAD), which is a tetrapeptide that blocks caspase-1 cleavage of the pro-IL-1β precursor and obstructs the secretion of the mature IL-1β from the cell (Gemma et al., 2007; Vojtech et al., 2012; Lin et al., 2018; Liang et al., 2019). Dark-adapted *albino;Tg*(*gfap:EGFP*)^*nt11*^ zebrafish were intravitreally injected with YVAD 24 h prior to the start of LT and every 24 h afterward. Retinas were collected at 36 h LT and immunolabelled for GFP and PCNA (Figures 3A–D). YVAD-treated retinas showed a significant decrease in the number of PCNA^+^ Müller glia relative to the DMSO (vehicle) control group (Figure 3E; DMSO: 23.3 ± 2.29; YVAD: 8.83 ± 1.30, *p* < 0.0001), suggesting that reducing the amount of mature IL-1β resulted in fewer proliferating Müller glia during LT.

To independently confirm this result, we also used morpholinos to knockdown IL-1β expression. Dark-adapted *albino;Tg*(*gfap:EGFP*)^*nt11*^ zebrafish were electroporated with either a Standard Control morpholino (S.C. MO) or *il-1β* MO (Figures 3F–I). Similar to the YVAD-treated zebrafish, the *il-1β* morphants possessed significantly fewer number of PCNA^+^ Müller glia relative to the S.C. morphants (Figure 3J; DMSO: 27.2 ± 2.48; *il-1β*: 7.34 ± 2.00, *p* < 0.0001). Because the *il-1β* morphant did not possess significantly fewer pyknotic nuclei at 36 h LT relative to the S.C. morphant (Supplementary Figures S1A, B, D, E), the reduced number of proliferating Müller glia in the *il-1β* morphant relative to the S.C. morphant was not due to decreased cell death. These data confirm that the pro-inflammatory cytokine IL-1β is required for Müller glia proliferation in light-damaged retinas.

**FIGURE 4:**
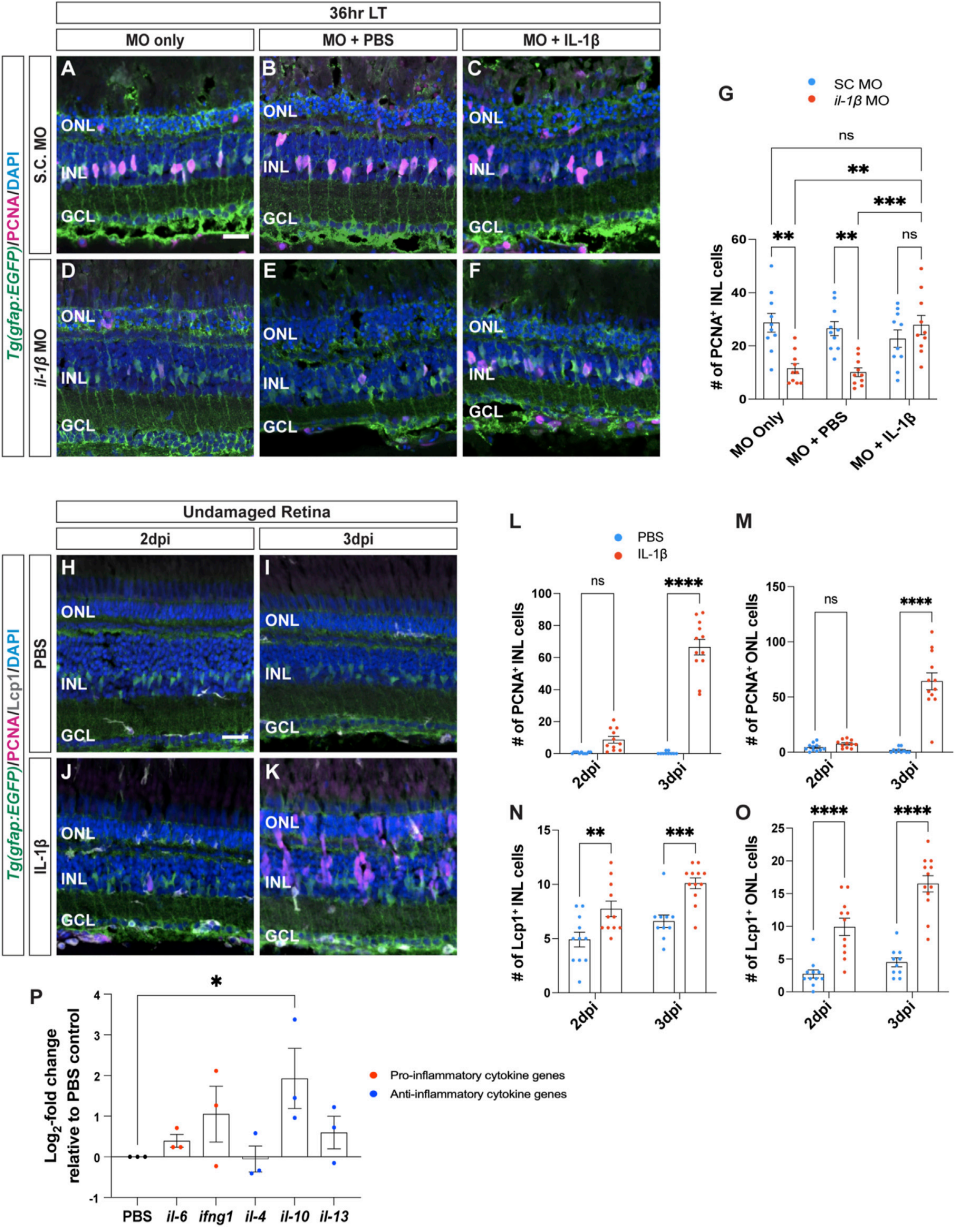
Recombinant IL-1β is sufficient to induce Müller glia proliferation in undamaged retinas. **(A–F)** Confocal images of *albino;Tg*(*gfap:EGFP*)^*nt11*^ retinas that were electroporated with Standard Control (S.C.; **(A–C)** and *il-1β* morpholinos **(D–F)** prior to the start of constant light treatment and intravitreally injected with either MO only **(A,D)**, PBS **(B,E)** or recombinant IL-1β protein **(C,F)**, and placed in LT. Retinal sections were collected at 36 h LT and immunostained to detect GFP (Müller glia, green) and PCNA (proliferating cells, magenta), with DAPI counterstain (nuclei, blue). **(G)** Quantification of the numbers of PCNA^+^ INL cells in *il-1β* morphants alone and injected with PBS (vehicle, blue circles) relative to S.C. morphants (red circles). Müller glia proliferation was rescued in *il-1β* morphants injected with IL-1β protein. **(H–K)** Confocal images of retinas from *albino;Tg*(*gfap:EGFP*)^*nt11*^ zebrafish that were injected with either PBS **(H,I)** or recombinant IL-1β protein **(J,K)** in undamaged retinas every 24 h. Retinal sections were collected at 2- and 3-days following the first injection (dpi) and immunolabeled for GFP (Müller glia, green) and PCNA (proliferating cells, magenta), with DAPI counterstain (nuclei, blue). **(L,M)** Quantifications showing the numbers of PCNA^+^ cells in the INL **(L)** and ONL **(M)** under different conditions described above. **(N,O)** Quantifications of the numbers of Lcp1^+^ cells in the INL **(N)** and ONL **(O)** under different conditions. **(P)** qRT-PCR analysis of pro-inflammatory (red circles) and anti-inflammatory (blue circles) cytokine gene expression profiles in undamaged retinas injected with IL-1β protein and collected at 3dpi. Data was normalized to 18 s rRNA reference gene and displayed as log_2-_fold change relative to the PBS (vehicle) control group. For the qRT-PCR, three independent replicates were performed with a pool of 6-7 dorsal retinas for each replicate. Cell count quantifications **(G,L–O)** were normalized to 300 μm along the length of the central-dorsal retina. Statistical analyses were performed using either a two-way ANOVA **(G,L–O)** or one-way ANOVA (P) both followed by Bonferroni’s *post hoc* test. Graphs represent the Mean ± SEM and n ≥ 10, *, *p* < 0.05, **, *p* < 0.01; ***, *p* < 0.001; ****, *p* < 0.0001; ns, no significance. ONL, outer nuclear layer; INL, inner nuclear layer; GCL, ganglion cell layer. Scale bar in A and H is 20 μm and is the same for **(B–F)** and **(I–K)**, respectively.

We next examined if the morpholino specifically knocked down IL-1β expression by testing if Müller glia proliferation in light-damaged *albino;Tg*(*gfap:EGFP*)^*nt11*^ *il-1β* morphant retinas was rescued by intravitreal injection of zebrafish IL-1β protein. Standard Control and *il-1β* morphant *albino;Tg*(*gfap:EGFP*)^*nt11*^ fish were intravitreally injected with recombinant zebrafish IL-1β protein every 24 h beginning at the start of constant light treatment (Figures 4A–F). As expected, fish that were electroporated with either *il-1β* MO alone or *il-1β* MO with PBS (vehicle) possessed significantly fewer PCNA^+^ Müller glia at 36 h LT relative to S.C. morphants (Figures 4A, B, D, E, G; S.C. MO alone: 28.70 ± 3.54, *il-1β* MO alone: 11.50 ± 1.88, *p* = 0.001; S.C. MO with PBS: 26.50 ± 2.62, *il-1β* MO with PBS: 10.1 ± 1.62, *p* = 0.002). In contrast, there was no significant difference in the number of PCNA^+^ Müller glia between the *il-1β* morphants injected with IL-1β protein and S.C. morphants injected with IL-1β protein (Figures 4C, F, G; S.C. MO with IL-1β protein: 22.70 ± 3.28, *il-1β* MO with IL-1β protein: 27.80 ± 3.61, *p* > 0.99). This data suggests that IL-1β protein rescued the Müller glia proliferation in *il-1β* morphants following light damage.

Because IL-1β is required for Müller glia proliferation, we examined if IL-1β is also sufficient to induce Müller glia proliferation in undamaged retinas. We intravitreally injected recombinant zebrafish IL-1β protein into undamaged *albino;Tg*(*gfap:EGFP*)^*nt11*^ zebrafish every 24 h and collected at 2- and 3-days following the initial injection (dpi) (Figures 4H–K). At 2dpi, there were no significant differences in the number of proliferating Müller glia between retinas injected with PBS and IL-1β (Figure 4L; PBS: 0.55 ± 0.16, IL-1β: 8.64 ± 2.06, *p* = 0.105). By 3dpi, retinas treated with IL-1β protein had significantly greater number of PCNA^+^ Müller glia and NPCs in the INL compared to the PBS (vehicle) control (Figure 4L; PBS: 0.40 ± 0.27, IL-1β: 66.50 ± 4.86, *p* < 0.0001) and in the ONL (Figure 4M; PBS: 1.7 ± 0.76, IL-1β: 64.33 ± 7.66, *p* < 0.0001). Furthermore, the number of Lcp1^+^ cells also significantly increased in retinas injected with IL-1β protein in both the INL and ONL at 2dpi (Figures 4N, O; PBS INL: 4.91 ± 0.68, IL-1β INL: 7.73 ± 0.72, *p* = 0.0054; PBS ONL: 2.73 ± 0.62, IL-1β ONL: 9.91 ± 1.32, *p* < 0.0001) and 3dpi (Figure N–O; PBS INL: 6.60 ± 0.58, IL-1β INL: 10.08 ± 0.50, *p* = 0.0006; PBS ONL: 4.50 ± 0.69, IL-1β ONL: 16.50 ± 1.25, *p* < 0.0001). However, we did not observe any signs of cell loss (TUNEL+ cells, Supplementary Figure S2) or pyknotic nuclei in the IL-1β-injected retinas (Figures 4J, K). This demonstrates that the pro-inflammatory cytokine IL-1β protein is sufficient to induce Müller glia proliferation even without retinal damage.

We next examined if injections of IL-1β affected the gene expression profiles of other inflammatory cytokines in the undamaged retinas. We performed qRT-PCR on *albino* zebrafish that were intravitreally injected with IL-1β protein for 3 days (Figure 4P). Only one of the five cytokines examined showed a significant increase in gene expression, *il-10* (Figure 4P; *il-10*: 2.90 ± 1.1, *p* = 0.0420) relative to the PBS control. Thus, not only is IL-1β necessary and sufficient to induce Müller glia proliferation following light damage, but it also likely activates the expression of *il-10* and possibly other cytokines.

### 3.4 Cytokine IL-10 is required for Müller glia proliferation following light damage

**FIGURE 5:**
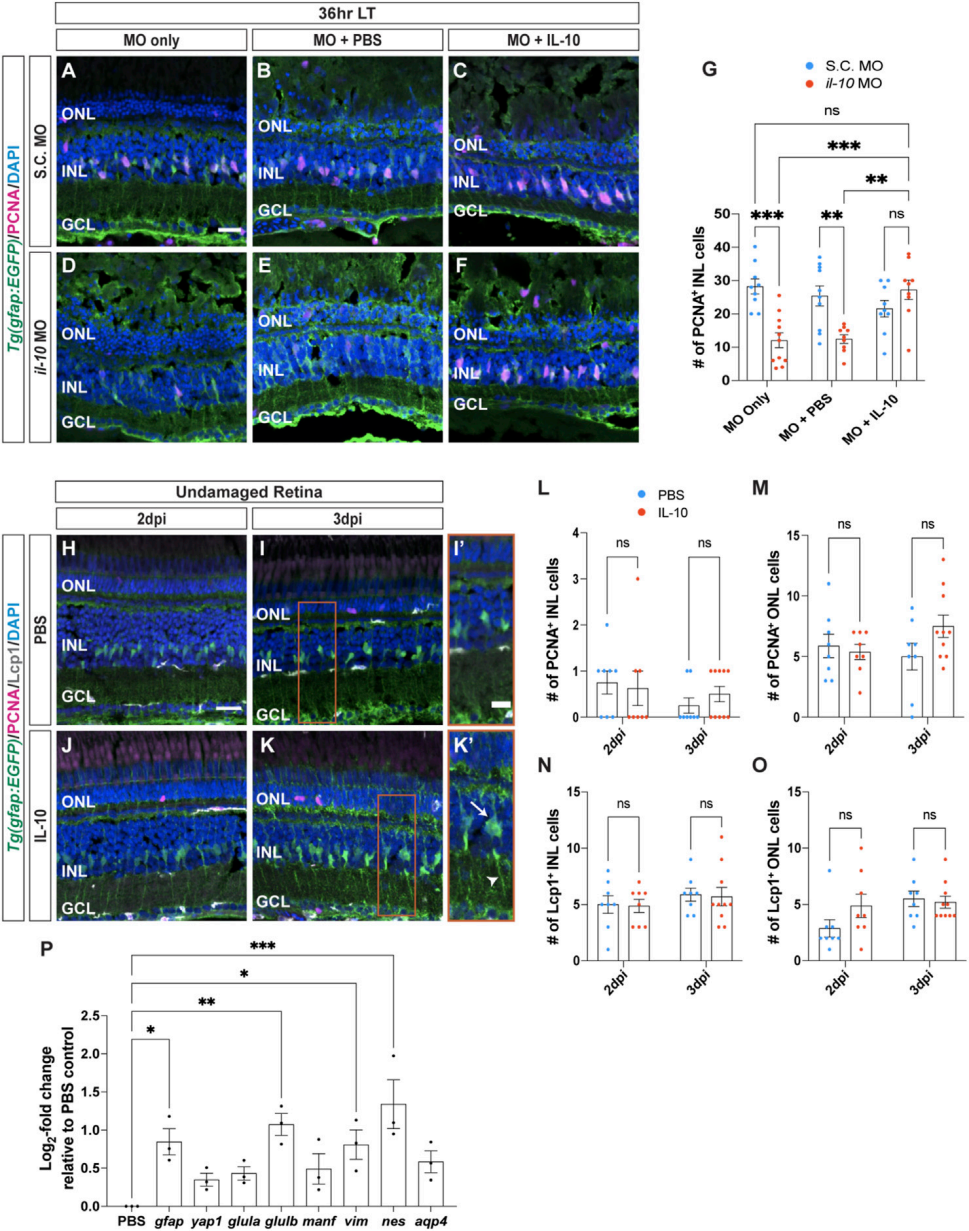
Cytokine IL-10 is necessary, but not sufficient, for Müller glia proliferation. **(A–F)** Confocal images of retinas from *albino;Tg*(*gfap:EGFP*)^*nt11*^ zebrafish that were electroporated with Standard Control (S.C.; **(A–C)** or *il-10* morpholinos **(D–F)** prior to the start of constant light treatment and intravitreally injected with either MO only **(A,D)**, PBS **(B,E)** or recombinant IL-10 protein **(C,F)**, and placed in LT. Retinal sections were collected at 36 h LT and immunolabeled for GFP (Müller glia, green) and PCNA (proliferating cells, magenta), with DAPI counterstain (nuclei, blue). **(G)** Quantifications of the numbers of PCNA^+^ INL cells under different conditions. Müller glia proliferation was rescued in *il-10* morphants injected with IL-10 protein. **(H–K)** Confocal images of *albino;Tg*(*gfap:EGFP*)^*nt11*^ retinas that were injected with either PBS **(H,I)** or recombinant IL-10 protein **(J,K)** in undamaged retinas every 24 h. Retinal sections were collected at 2- and 3-days following the first injection (dpi) and immunolabeled to detect GFP (Müller glia, green) and PCNA (proliferating cells, magenta), with DAPI counterstain (nuclei, blue). Red boxes in I and K were magnified to better portray the EGFP signal in the hypertrophied Müller glia **(I’,K’)**. Arrow and arrowhead mark a hypertrophied cell body and process, respectively. **(L–O)** Quantifications of the numbers of PCNA^+^ cells (L, M) and Lcp1^+^ cells **(N,O)** in the INL **(L,N)** and ONL **(M,O)**. **(P)** qRT-PCR analysis of gliotic-associated gene expression in undamaged retinas injected with IL-10 protein and collected at 3dpi. Data was normalized to 18 s rRNA reference gene and displayed as log_2-_fold change relative to the PBS (vehicle) control group. For the qRT-PCR, three independent replicates were performed with a pool of 6-7 dorsal retinas for each replicate. Cell count quantifications **(G,L–O)** were normalized to 300 μm along the length of the central-dorsal retina. Statistical analyses were performed using either a two-way ANOVA **(G,L–O)** or one-way ANOVA **(P)** both followed by Bonferroni’s *post hoc* test. Graphs represent the Mean ± SEM and n ≥ 8. *, *p* < 0.05, **, *p* < 0.01; ***, *p* < 0.001; ns, no significance. ONL, outer nuclear layer; INL, inner nuclear layer; GCL, ganglion cell layer. Scale bars in **(A,H)** are 20 μm and are the same for **(B–F)** and **(I–K)**, respectively. Scale bar in **(I′)** is 10 μm and is the same for **(K’)**.

Cytokine IL-10 often possesses anti-inflammatory effects that can regulate the expression of pro-inflammatory cytokines released by immune cells, such as microglia (O’Garra and Vieira, 2007; Lobo-Silva et al., 2017; Shemer et al., 2020). To determine if IL-10 is required for Müller glia proliferation following light damage, Standard Control and *il-10* morpholinos were intravitreally injected alone or with PBS every 24 h until 36 h LT (Figures 5A–F). Similar to the *il-1β* morphants*,* the *il-10* morphants had significantly fewer proliferating Müller glia in the INL compared to the S.C. morphants either MO alone or with PBS groups (Figures 5A, B, D, E, G; S.C. MO alone: 28 ± 2.56, *il-10* MO alone: 12.07 ± 2.24, *p* = 0.0002; S.C. MO with PBS: 25.40 ± 3.00, *il-10* MO with PBS: 12.44 ± 1.29, *p* = 0.007). Because the *il-10* morphant did not possess significantly fewer pyknotic nuclei at 36 h LT relative to the S.C. morphant (Supplementary Figures S1A, B, D, E), the reduced number of proliferating Müller glia in the *il-10* morphant relative to the S.C. morphant was not due to decreased cell death. Additionally, Müller glia proliferation was rescued when IL-10 protein was intravitreally injected every 24 h into the *il-10* morphants (Figures 5C, F, G; S.C. MO with IL-10: 21.56 ± 2.45. *il-10* MO with IL-10: 27.22 ± 2.91, *p* > 0.99). This demonstrated that the *il-10* morpholino specifically knocked down IL-10 expression, which is required for Müller glia proliferation following light damage.

To determine if IL-10 is sufficient to induce Müller glia proliferation, IL-10 protein was intravitreally injected into undamaged retinas every 24 h and collected at 2dpi and 3dpi (Figures 5H–K). There was no significant difference in the number of PCNA^+^ Müller glia between the PBS and IL-10 protein injected groups in either the INL or ONL at 2dpi (Figures 5L, M; PBS INL: 0.75 ± 0.25, IL-10 INL: 0.63 ± 0.38, *p* > 0.99; PBS ONL: 5.88 ± 0.97, IL-10 ONL: 5.38 ± 0.63, *p* > 0.99) and 3dpi (Figures 5L, M; PBS INL: 0.25 ± 0.16, IL-10 INL: 0.50 ± 0.17, *p* > 0.99; PBS ONL: 5.00 ± 1.10, IL-10 ONL: 7.50 ± 0.92, *p* > 0.99). There were also no significant difference in the number of Lcp1^+^ cells relative to the PBS controls in either the INL or ONL at 2dpi (Figures 5N, O; PBS INL: 5.00 ± 0.78, IL-10 INL: 4.88 ± 0.58, *p* > 0.99; PBS ONL: 2.88 ± 0.77, IL-10 ONL: 4.88 ± 1.04, *p* > 0.99) and at 3dpi (Figures 5N, O; PBS INL: 5.88 ± 0.58, IL-10 INL: 5.70 ± 0.82, *p* > 0.99; PBS ONL: 5.50 ± 0.68, IL-10 ONL: 5.20 ± 0.53, *p* > 0.99). Thus, IL-10 is necessary, but not sufficient to induce Müller glia proliferation.

Upon closer examination, undamaged retinas injected with IL-10 possessed Müller glia that displayed a gliotic morphology after 3dpi, with hypertrophied soma and processes (Figures 5I’, K’, arrows and arrowhead, respectively). To confirm that these Müller glia were gliotic, we performed qRT-PCR on various gliotic markers including *gfap, yap1, glutamine synthase* (*glula/b*)*, mesencephalic astrocyte-derived neurotrophic factor* (*manf*)*, vimentin* (*vim*)*, nestin* (*nes*)*,* and *aquaporin 4* (*aqp4*) (Figure 5P). The *gfap*, *glulb*, *vim*, and *nes* gliotic marker genes were significantly upregulated in expression in the IL-10 injected retinas relative to the PBS (vehicle) control group after 3dpi (Figure 5P; *gfap*: 0.85 ± 0.30, *p* = 0.021; *yap1*: 0.35 ± 0.15, *p* > 0.99; *glula:* 0.43 ± 0.15, *p* = 0.73; *glulb*: 1.10 ± 0.25, *p* = 0.0026; *manf*: 0.49 ± 0.34, *p* = 0.46; v*im*: 0.81 ± 0.33, *p* = 0.030*; nes*: 1.3 ± 0.55, *p* = 0.0002; *aqp4*: 0.58 ± 0.25, *p* = 0.22). Thus, IL-10 is necessary for Müller glia proliferation in damaged retinas, while injection of IL-10 in undamaged retinas stimulated the Müller glia to exhibit a gliotic phenotype.

### 3.5 IL-1β can stimulate Müller glia proliferation, independent of IL-10 expression in undamaged retinas

**FIGURE 6:**
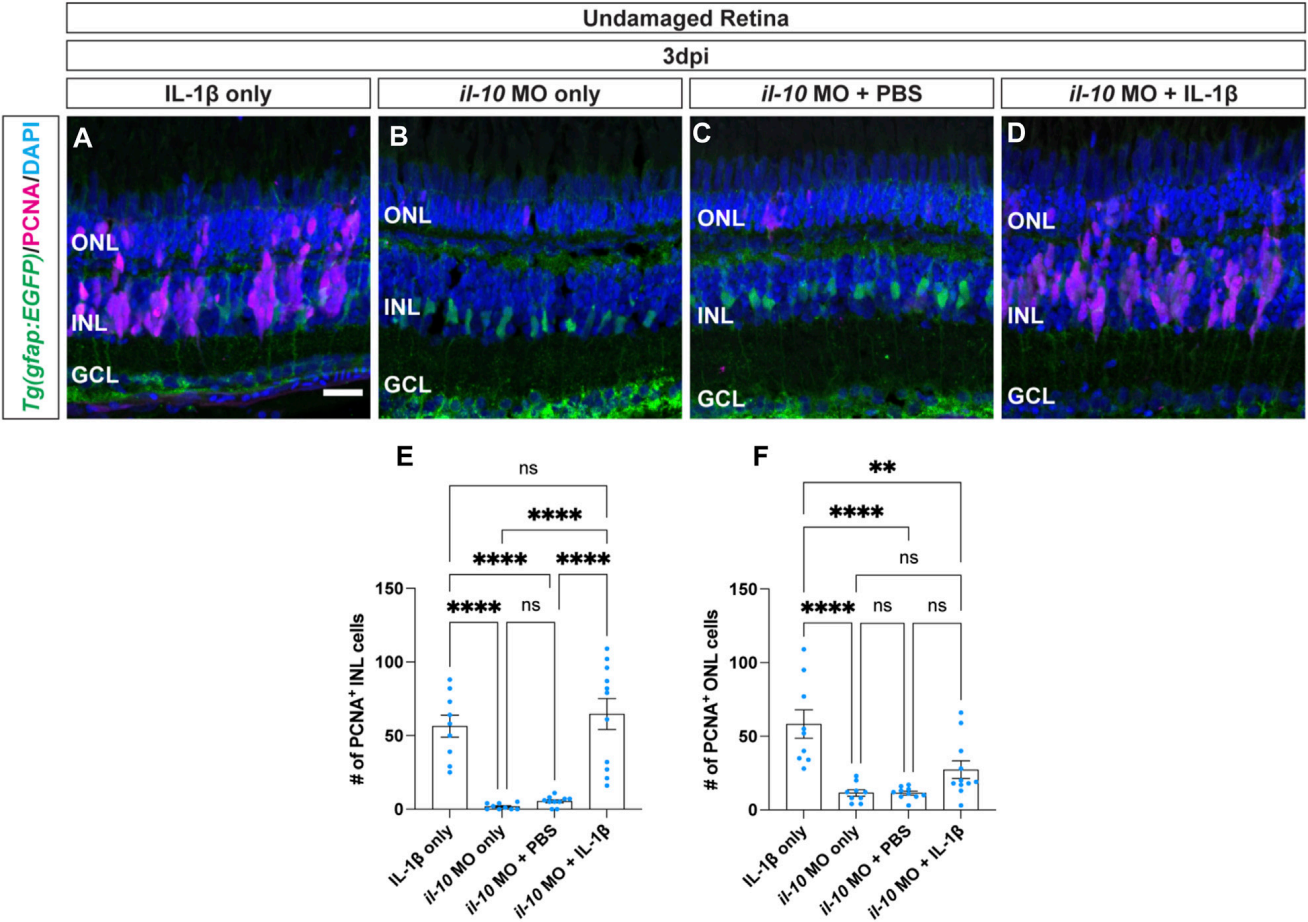
Cytokine IL-1β induction of Müller glia proliferation does not require IL-10. **(A–D)** Confocal images of undamaged *albino;Tg*(*gfap:EGFP*)^*nt11*^ zebrafish retinas that were intravitreally injected with IL-1β alone **(A)**, or electroporated with *il-10* morpholino and intravitreally injected with either MO only **(B)**, PBS **(C)**, or IL-1β protein **(D)**. Sections were collected at 3 days post-injection (dpi) and immunostained for GFP (Müller glia, green) and PCNA (proliferating cells, magenta), with DAPI counterstain (nuclei, blue). **(E)** Quantifications of the numbers of PCNA^+^ INL cells under different conditions. **(F)** Quantifications of the numbers of PCNA^+^ ONL cells under different conditions. Quantifications were normalized to 300 μm along the central-dorsal retina. Statistical analyses were performed using one-way ANOVA followed by Tukey’s *post hoc* test. Mean ± SEM and n ≥ 9, **, *p* < 0.01; ****, *p* < 0.0001; ns, not significant. ONL, outer nuclear layer, INL, inner nuclear layer, GCL, ganglion cell layer. Scale bar in A is 20 μm and is the same for **(B–D)**.

We demonstrated that *il-1β* expression is induced before *il-10* and both genes are required for Müller glia proliferation in light-damaged retinas. Additionally, injection of IL-1β into undamaged retinas induced *il-10* expression and stimulated Müller glia proliferation. However, injection of IL-10 into the undamaged retina did not induce Müller glia proliferation. Thus, IL-1β may be required to induce *il-10* expression in the undamaged retina in order to stimulate Müller glia and/or NPC proliferation. To test this hypothesis, we injected IL-1β protein into undamaged retinas with and without the *il-10* morpholino. The *albino;Tg*(*gfap:EGFP*)^*nt11*^ zebrafish were electroporated with *il-10* morpholino at 0dpi and then injected with recombinant zebrafish IL-1β protein every 24 h and collected at 3dpi (Figures 6B–D). These were compared to undamaged *albino;Tg*(*gfap:EGFP*)^*nt11*^ zebrafish that were injected with only recombinant zebrafish IL-1β protein every 24 h and collected at 3dpi (Figure 6A). The *albino;Tg*(*gfap:EGFP*)^*nt11*^ fish that were only injected with IL-1β possessed a large number of PCNA^+^ Müller glia and NPCs at 3dpi in both the INL and ONL (Figures 6A, E, F; INL: 56.44 ± 7.52, ONL: 58 ± 9.65). The *il-10* morphants alone and the *il-10* morphants injected with PBS (vehicle) control possessed very low numbers of PCNA^+^ Müller glia and NPCs in the INL (Figures 6B, C, E; *il-10* MO:1.78 ± 0.68; *il-10* MO with PBS: 5.40 ± 1.07) and ONL (Figures 6B, C, F; *il-10* MO: 11.56 ± 2.19, *il-10* MO with PBS: 11.40 ± 1.27). However, *il-10* morphants injected with IL-1β protein possessed similar numbers of PCNA^+^ Müller glia and NPCs to zebrafish injected with only IL-1β protein in the INL (Figures 6A, D, E; IL-1β: 56.44 ± 7.52, *il-10* MO with IL-1β: 64.73 ± 10.50, *p* > 0.99), but not in the ONL (Figures 6A, D, F; IL-1β: 58.33 ± 9.65, *il-10* MO with IL-1β: 27.36 ± 5.92, *p* = 0.0027). This demonstrates that IL-1β induces Müller glia proliferation independent of IL-10 expression in the undamaged retina.

## 4 Discussion

In recent years, it was demonstrated that inflammation plays a critical role in regulating regeneration of zebrafish retinal neurons (Nagashima and Hitchcock, 2021). In this study, we focused on the expression of cytokines and their role in inducing Müller glia proliferation following light damage at the onset of retinal regeneration in adult zebrafish. We found that a subset of cytokines, which are usually known to induce inflammation, increases in expression shortly after the start of light-induced retinal damage. As their expression begins to decrease, a subset of cytokines, which are often associated with inhibiting inflammation, increases in expression, suggesting that this dynamic temporal pattern of expression is necessary for Müller glia proliferation and regeneration of retinal neurons following light damage. We then demonstrated that expression of the pro-inflammatory cytokine IL-1β is both necessary and sufficient to induce Müller glia proliferation. In contrast, expression of the cytokine IL-10 is necessary, but not sufficient, for Müller glia proliferation. Thus, the expression of various cytokines plays a key role in stimulating Müller glia proliferation following damage in the adult zebrafish retina.

We examined the expression of a subset of cytokines in light-damaged retinas. The expression of three cytokine genes, which usually exhibit pro-inflammatory effects: *il-1β, il-6,* and *ifng1* were upregulated shortly after starting the constant light treatment, while three additional cytokine genes, which often possess anti-inflammatory effects: *il-4, il-10,* and *il-13* were delayed before increasing their expression. These findings aligned with our previous study, which revealed differential expression of inflammatory genes following NMDA-damage in the chronic *gosh* cone degeneration mutant retinas (Iribarne and Hyde, 2022). This dynamic expression of cytokines can be generated in several different ways, with one possibility being that one or more pro-inflammatory cytokines induce the expression of cytokines, which are commonly associated with inhibiting inflammation. Consistent with this model, we found that intravitreal injection of recombinant IL-1β was sufficient to stimulate *il-10* expression in undamaged retinas. This is similar to what was observed in rodent models of experimental autoimmune encephalomyelitis (EAE), where expression of IL-1β activated and translocated NF-ΚB to the nucleus and induced expression of inflammatory cytokines, IL-1β and TNF-α, as well as the IL-10 (Tomczak et al., 2006; Lawrence, 2009; Goldmann et al., 2013; Zhou et al., 2020). Whether other inflammatory cytokines induce the expression of additional cytokines associated with inhibiting inflammation, or if other regulatory mechanisms are at work here, remains to be determined.

**FIGURE 7:**
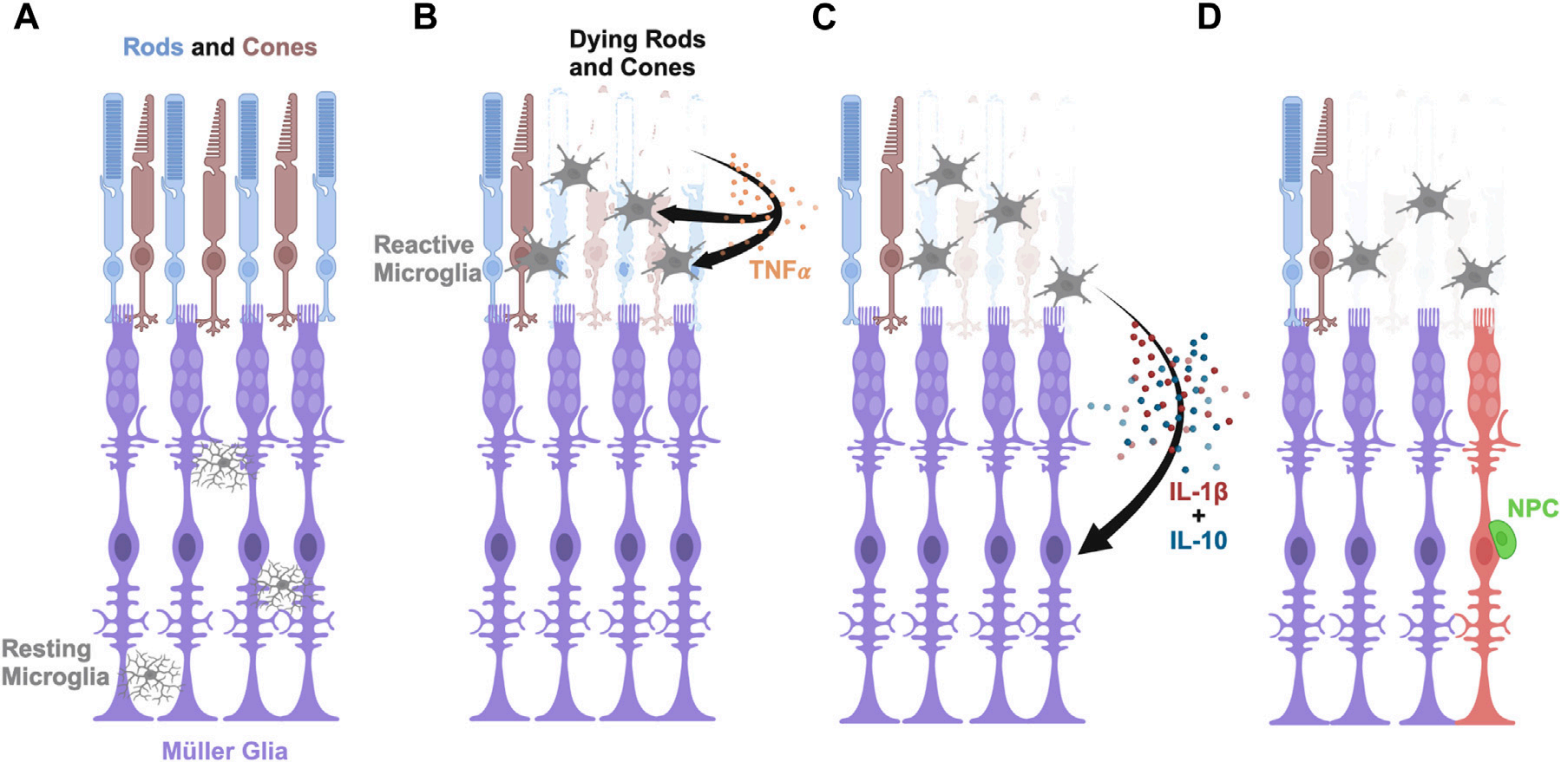
Model of pro-inflammatory cytokine IL-1β and anti-inflammatory cytokine IL-10 inducing Müller glia proliferation in light-damaged retinas. **(A)** Schematic depicting rod and cone photoreceptors (blue and brown, respectively), Müller glia (purple), and resting microglia (gray) in an undamaged retina. **(B)** Upon light damage, the resting microglia move from the locations in the plexiform layers to the outer retina where they become activated and begin phagocytosing dying rods and cones. The dying photoreceptors express TNFα. **(C)** The activated microglia express IL-1β and IL-10 in a dynamic fashion that signal the Müller glia to reprogram. **(D)** The reprogrammed Müller glia divide asymmetrically to produce a neuronal progenitor cell (NPC, green), which will continue to proliferate and differentiate into the missing retinal neurons.

Our finding that IL-1β is necessary to induce Müller glia proliferation in the damaged retina and sufficient to stimulate a similar response in undamaged retinas is similar to the ability of the inflammatory cytokine TNF-α inducing Müller glia proliferation in undamaged retinas (Nelson et al., 2013). However, these two inflammatory cytokines differ in the source of their initial expression, with TNF-α initially being expressed in dying retinal neurons (Nelson et al., 2013) and IL-1β expressed in microglia. However, *tnfa* is also expressed in microglia, but peaks in only 14% of the microglia at 20 h LT (Hoang et al., 2020). It is unlikely that both IL-1β and TNF-α induce Müller glia proliferation through redundant pathways because morpholino-mediated knockdown of either IL-1β or TNF-α is sufficient to significantly reduce Müller glia proliferation. Additionally, it is unlikely that they each stimulate an independent pathway because intravitreal injection of either IL-1β or TNF-α is sufficient to stimulate Müller glia proliferation. It is more likely that TNF-α expression from the dying retinal neurons stimulates the microglia, which in turn expresses IL-1β, and possibly also induces *tnfa* expression in the microglia, to induce the reprogramming and proliferation of the Müller glia (Figure 7). Alternatively, TNF-α stimulates both the microglia and Müller glia, although at a low level, and the stimulation of the microglia induces IL-1β expression that amplifies the induction of the Müller glia to reprogram and proliferate. In either case, IL-1β would function downstream of TNF-α in stimulating the maximal number of Müller glia to reprogram and proliferate.

We also examined the potential role of the IL-10 on Müller glia proliferation in the light-damaged adult zebrafish retina. Previous studies examined the role of anti-inflammatory cytokines, like IL-10, on ocular diseases, but the specific role of IL-10 on Müller glia reprogramming and proliferation has not been previously examined (Nakamura et al., 2015; Eastlake et al., 2016; Nikoopour et al., 2019). We found that IL-10 was necessary for Müller glia proliferation in light-damaged retinas, but it was not sufficient to induce Müller glia proliferation in undamaged retinas. Further, we demonstrated that intravitreal injection of IL-1β into undamaged retinas was sufficient to significantly induce IL-10 expression. Taken together, we propose that TNFα is produced by the dying photoreceptors (Nelson et al., 2013), which stimulates the expression of *il-1β* and *il-10* in the microglia (Figures 7B, C). IL-1β further induces *il-10* expression, which is required for Müller glia reprogramming and proliferation in the adult zebrafish retina to produce neuronal progenitor cells (Figure 7D). Thus, Müller glia proliferation in response to light damage requires the expression of both pro-inflammatory and anti-inflammatory cytokines. This would account for both IL-1β and IL-10 being required for Müller glia proliferation in the light-damaged retina, the ability of intravitreal injection of IL-1β to induce Müller glia proliferation in undamaged retinas (due to IL-1β inducing *il-10* expression), and the inability of intravitreal injection of IL-10 to induce Müller glia proliferation in the undamaged retinas (due to the absence of increased *il-1β* expression).

Because IL-10 is necessary for Müller glia proliferation, it was unexpected that intravitreal injection of IL-10 into undamaged retinas induced Müller glia to enter a gliotic-like state rather than either stimulating Müller glia proliferation or having no discernible effect. It was previously demonstrated that Müller glia enter a transient gliotic state before reprogramming and proliferation, with a gliotic cell morphology, including cell hypertrophy, as well as increased expression of several gliotic genes (Thomas et al., 2018; Hoang et al., 2020). However, *il-10* expression peaks several hours after the peak of *il-1β* expression in light-damaged retinas (Figure 2A). There are two possible explanations for how IL-10 could induce a gliotic state in undamaged retinas. First, there is a small, but significant increase in *il-10* expression that coincides with increasing *il-1β* expression (Figure 2A). It is possible that a low level of IL-10 expression leads to the transient gliotic state before increasing IL-1β expression induces the Müller glia to reprogram and reenter the cell cycle. In this model, the application of a high level of IL-10 to the undamaged retina, without increased IL-1β expression, would lead to gliosis rather than Müller glia proliferation. In the second model, IL-10 does not induce the transient gliotic state in the light-damaged retina. However, in the undamaged retina, where regeneration signals (TNFα and IL-1β) are absent and IL-10 is exogenously applied, the IL-10 protein may induce the gliotic state through an unrelated mechanism. Differentiating between these two models will require precisely altering the dynamic timing and expression levels of these two cytokines.

What this model fails to account for is that intravitreal injection of IL-1β is sufficient to induce Müller glia proliferation in undamaged *il-10* morphant retinas. Based on our model, intravitreal injection of IL-1β would require increased *il-10* expression to stimulate Müller glia proliferation. It is possible that the morpholino-mediated knockdown does not entirely abolish *il-10* expression. There are numerous examples where the morpholino does not entirely abolish expression of the target protein (Nelson et al., 2012; Gorsuch et al., 2017). In this case, the intravitreal injection of IL-1β could have been sufficiently large that it induced a greater level of expression of *il-10* than present in the light-damaged retina, such that it induced a level of *il-10* transcript was large enough to allow a sufficient amount of IL-10 protein even though the morpholino blocked a large amount of Il-10 protein translation. Alternatively, the intravitreal injection of IL-1β into undamaged retinas was greater than what is normally present in the light-damaged retina and this unusually high level of IL-1β had altered effects on other cytokines and this resulted in the nontraditional mechanism to induce Müller glia proliferation. Given the dynamic nature of cytokine expression following light damage (Figure 2A), it is possible that precise timing and level of individual cytokine expression are the critical components in Müller glia proliferation.

Although this study focused on the roles of two major cytokines, IL-1β and IL-10 in the light-damaged adult zebrafish retina, we cannot overlook the potential contributions of other cytokines involved in Müller glia proliferation and neuronal regeneration. IL-6 family cytokines, along with p-Stat3 signaling, were shown to stimulate zebrafish Müller glia reprogramming (Zhao et al., 2014). Similarly, TNF-α is required for Müller glia proliferation in the zebrafish retina, although it is produced in dying retinal neurons (Nelson et al., 2013). Outside the retina, IL-4 suppressed inflammation following zebrafish gill tissue damage (Bottiglione et al., 2020), while IL-10 and IL-4 signaling worked synergistically together to induce synaptogenesis in sensory hair cells and motor neurons in zebrafish (Denans et al., 2022). As numerous signaling events take place during damage and initiation of neuronal regeneration, the dynamic timing and interaction between these cytokines and both neuronal and non-neuronal cells found in the retina may play a critical role in determining the successful regeneration of neurons in the zebrafish retina following injury. While other cytokines and signaling molecules are likely involved in this mechanism to initiate and regulate retinal regeneration, the identification of IL-1β and IL-10 as being necessary for Müller glia proliferation in the regeneration process provides a strong foundation to build future investigations.

## Data Availability

The original contributions presented in the study are included in the article/Supplementary Material, further inquiries can be directed to the corresponding author. All scRNA-seq data and source codes are available at GitHub https://github.com/jiewwwang/Single-cell-retinal-regeneration. The scRNA-seq data can be queried interactively at https://proteinpaint.stjude.org/F/2019.retina.scRNA.html.

## Supplementary material

SUPPLEMENTARY FIGURE S1 Morpholino-mediated knockdown of either IL-1β or IL-10 does not significantly alter the extent of photoreceptor cell death following light damage. **(A–C)** Confocal images of *albino;Tg(gfap:EGFP)* ^*nt11*^ zebrafish retinas that were electroporated with either Standard Control S.C; **(A)**, *il-1β* **(B)**, or *il-10* **(C)** morpholinos prior to the start of constant light treatment. Retinas were collected at 36hr LT and counterstained with DAPI nuclei, blue; **(A–C)**. Quantifications showing the number of pyknotic cells in the INL **(D)** and ONL **(E)** at 36hr LT. Cell counts were normalized to 300 μm along the length of the central-dorsal retina. Statistical analyses were performed using one-way ANOVA followed by Bonferroni’s post-hoc test. Graphs represent the Mean ± SEM and n ≥ 5. ns represents no significance. ONL, outer nuclear layer; INL, inner nuclear layer; GCL, ganglion cell layer. Scale Bar in A is 20 μm and is the same for **(B,C)**.

SUPPLEMENTARY FIGURE S2 Intravitreal injection of IL-1β protein does not induce cell death in the undamaged retina. **(A–D)** Confocal images of *albino;Tg(gfap:EGFP)* ^*nt11*^ zebrafish retinas that were intravitreally injected with recombinant IL-1β protein in the undamaged retinas every 24 hours for 3 days. Sections were collected at 2- and 3- days following the first injection (dpi). TUNEL assay was performed to identify apoptotic cells (magenta) and retinal sections were immunostained with GFP (Müller glia, green) and counterstained with DAPI (nuclei, blue). **(E,F)** Quantifications showing the number of TUNEL^+^ cells in the INL **(E)** and ONL **(F)** at 2- and 3-dpi. Cell counts were normalized to 300 μm along the length of the central-dorsal retina. Statistical analyses were performed using two-way ANOVA followed by Bonferroni’s *post-hoc* test. Mean ± SEM, n ≥ 5. ns no significance. ONL, outer nuclear layer; INL, inner nuclear layer; GCL, ganglion cell layer. Scale bar in A is 20 μm and is the same for **(B–D)**.
