# Supplementary figures and images for: Cytokines IL-1β and IL-10 are required for Müller glia proliferation following light damage in the adult zebrafish retina
Source: Front Cell Dev Biol. 2024 Jun 13;12:1406330. doi: 10.3389/fcell.2024.1406330 (PMC11208712; doi:10.3389/fcell.2024.1406330)

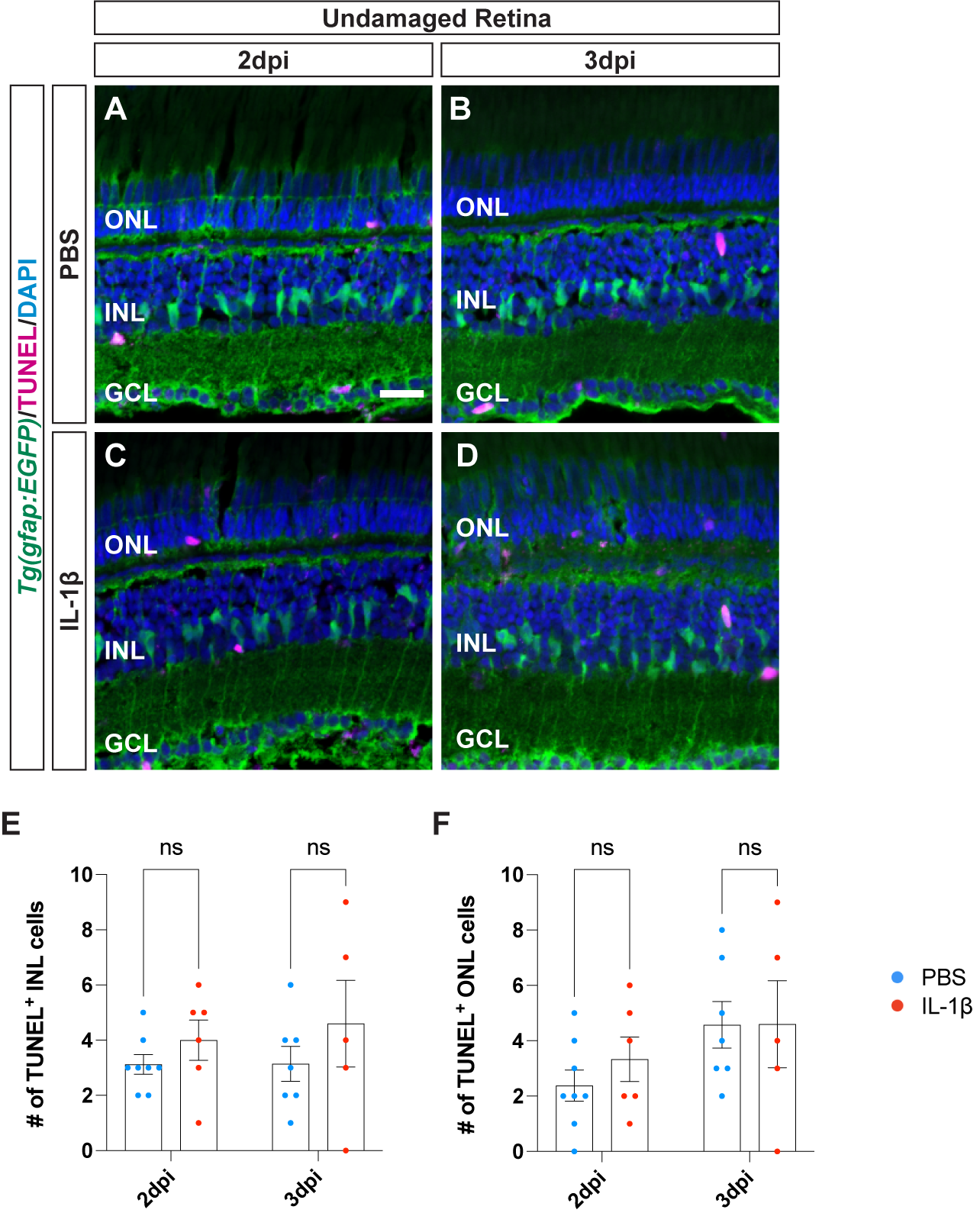

Supplement: Supplementary file 1 [file Image2.TIF]

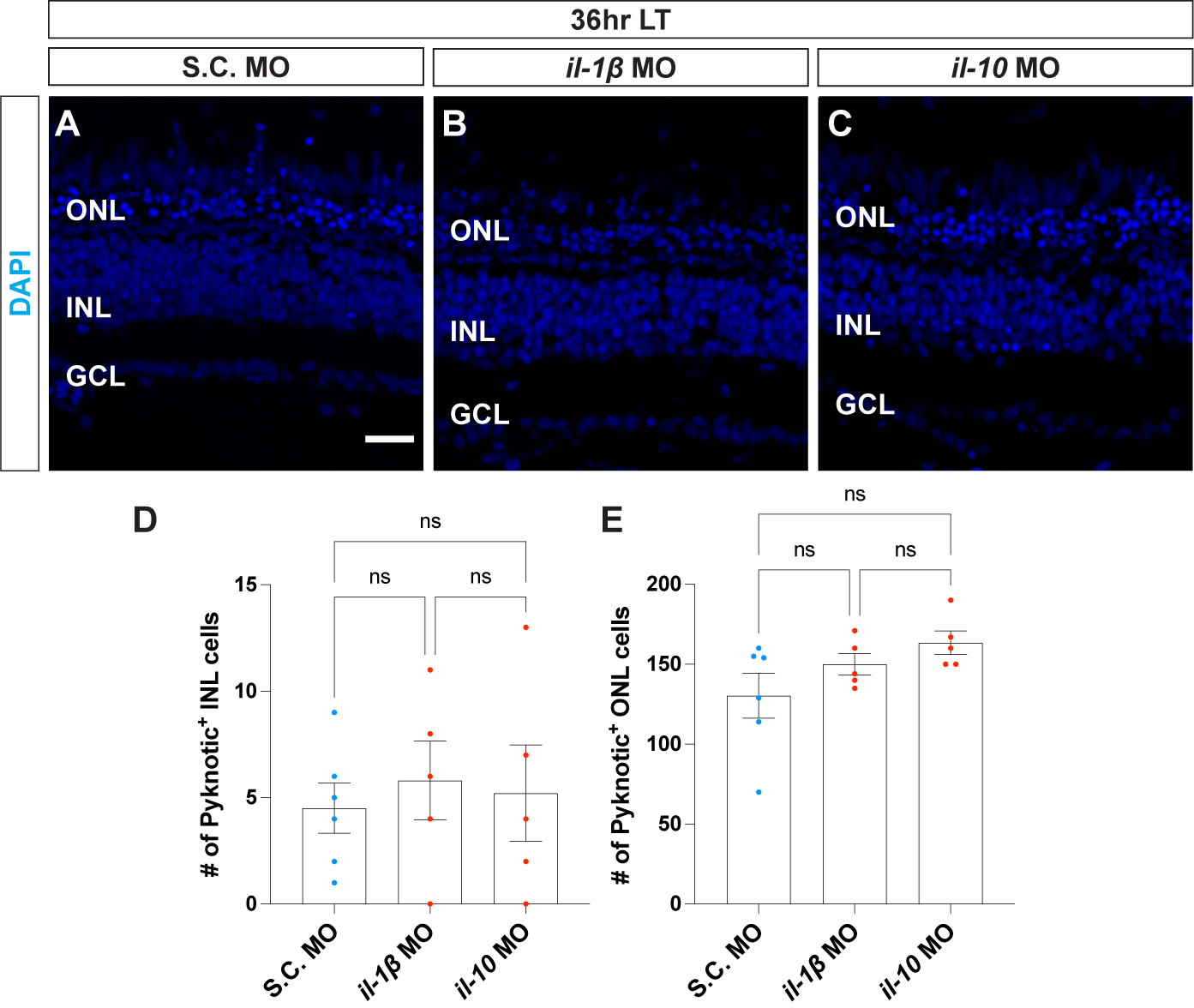

Supplement: Supplementary file 2 [file Image1.TIF]
